# Psychosocial Outcomes in Telemedicine and Long‐Acting Incretin‐Specific Behavioral Intervention

**DOI:** 10.1002/osp4.70149

**Published:** 2026-05-20

**Authors:** Leslie J. Heinberg, Alexandra M. Lee, Gary D. Foster, Alex Zajichek, Spencer Nadolsky, Amy DiVita, Daniel Rotroff, Michelle I. Cardel

**Affiliations:** ^1^ Department of Psychiatry and Psychology Cleveland Clinic Lerner College of Medicine Cleveland Ohio USA; ^2^ WW International Inc New York New York USA; ^3^ Weight and Eating Disorders Program Perelman School of Medicine University of Pennsylvania Philadelphia Pennsylvania USA; ^4^ Department of Quantitative Health Sciences Cleveland Clinic Cleveland Ohio USA; ^5^ Vineyard Health Livermore California USA; ^6^ Department of Psychiatry and Psychology Cleveland Clinic Cleveland Ohio USA; ^7^ Lerner Research Institute Cleveland Clinic Cleveland Ohio USA; ^8^ Department Health Outcomes & Biomedical Informatics University of Florida College of Medicine Gainesville Florida USA; ^9^ Department of Pharmaceutical Outcomes & Policy University of Florida College of Pharmacy Gainesville Florida USA; ^10^ Center for Integrative Cardiovascular & Metabolic Disease University of Florida Gainesville Florida USA

**Keywords:** anti‐obesity medication, behavior, intervention, lifestyle, telemedicine

## Abstract

**Background:**

Newer generation anti‐obesity medications such as semaglutide and tirzepatide are highly efficacious, resulting in a proliferation of telehealth prescribers. Few, however, augment medication with a behavioral intervention, and little research has examined the impact of these medications on psychosocial variables.

**Objective:**

This study evaluated the efficacy of a telehealth provision of semaglutide/tirzepatide with a virtual behavioral program on psychosocial outcomes at 12 and 24 weeks.

**Methods:**

In this single‐arm pragmatic trial, 180 participants (M age = 44.1; 91% female; 81% white; M weight = 102.6 kgs) were recruited from a telemedicine obesity program (WeightWatchers Clinic) and offered an adjunctive virtual behavioral program tailored for patients on long‐acting incretin therapy. Participants completed the PHQ‐8 measuring depression, Perceived Stress Scale, Self‐Compassion Scale, WHO‐5 measuring well‐being, Weight Bias Internalization Scale‐2F and Impact of Weight on Quality of Life‐Lite at baseline, 12 and 24 weeks. Wilcoxon signed‐rank tests were conducted to evaluate outcome differences. *p* values were adjusted using a False Discovery Rate approach. Intent‐to‐treat analysis was performed using last observation carried forward (LOCF).

**Results:**

There were significant improvements from median baseline to 24 weeks in measures of depression (−2), perceived stress (−3), well‐being (3), weight bias internalization (−0.6), and impact of weight on quality of life (−18). All *p*'s < 0.001 in the baseline to 24‐week analyses with similar results shown at 12 weeks.

**Conclusions:**

Virtually delivered obesity treatment combining telemedicine and a long‐acting incretin‐specific behavioral program demonstrated clinically significant improvements in psychosocial outcomes over 24 weeks.

## 1 Introduction

Recently, a new generation of anti‐obesity medications (AOM's) approved for long‐term obesity treatment have demonstrated efficacy approaching that of metabolic and bariatric surgical procedures [1, 2]. Semaglutide was FDA‐approved in 2021 for chronic weight management in adults with overweight or obesity. It is a glucagon‐like peptide‐1 (GLP‐1) receptor agonist that has demonstrated 15% body weight loss at 68 weeks [1]. Tirzepatide was FDA approved for weight loss in 2023. It has agonist activity at both the GIP and GLP‐1 receptors [2], with a 21% body weight loss at 72 weeks [2].

Most clinical trials examining the efficacy of these AOMs have focused on weight and reduction in co‐morbidities (e.g., blood pressure, HbA1c). Semaglutide was shown to have superiority over placebo in improving health‐related and weight‐related quality of life in the STEP 1–4 Phase 3a trial [3]. Similarly, data from the SURMOUNT trial evaluating tirzepatide included an obesity‐specific quality of life measure and demonstrated significant improvements in the physical function and psychosocial component scores [4]. Depression scores have also been shown to improve with the use of these AOMs [5], but there have been mixed findings on these medications and increased suicidal ideation [6, 7, 8, 9]. However, little is known about other psychosocial outcomes, including weight bias internalization, self‐compassion, perceived stress and general well‐being.

Furthermore, few data exist about these incretin‐based AOMs in treatment settings that are not highly structured by research protocols. This limits clinical practice recommendations as patient demand is very high outside research settings. Indeed, 59% of individuals who are trying to lose weight are interested in trying AOMs [10]. Additionally, increased patient demand and preference for virtual visits along with limited access to obesity specialty care have driven the demand for telemedicine obesity treatment [11, 12]. Overall, the data on these approaches is minimal with extremely limited inclusion of psychosocial outcomes. A recent observational, retrospective study demonstrated the effectiveness of telehealth provision of AOMs with weight loss outcomes consistent with published clinical trials [13]. Recently, we conducted a prospective, single‐arm trial of a telemedicine obesity treatment with semaglutide or tirzepatide coupled with a behavioral program tailored to those on long‐acting incretin therapy and found significant improvements in weight, blood pressure, dietary quality and lifestyle behaviors [14]. However, studies have yet to examine psychosocial outcomes in this class of medications when delivered via telehealth delivery and when it includes an appropriately matched virtual behavioral intervention.

Thus, the purpose of this study was to prospectively examine mood, stress, well‐being, self‐compassion, internalized weight bias and quality of life within a weight loss trial that utilized a widely available telemedicine offering. Additionally, the telemedicine program in the current study included a virtual adjunctive behavioral program tailored for those on long‐acting incretin therapy that focused on nutrition, physical activity, support and behavioral principles hypothesized to impact the non‐weight outcomes. The primary objective was to assess the impact of telemedicine‐delivered semaglutide/tirzepatide when combined with a virtual tailored adjunctive behavioral program on psychosocial variables at 24 weeks. Secondary analyses focused on intermediate effects at 12 weeks.

## 2 Methods

### 2.1 Participant Population

Participants in the current one‐arm prospective trial consisted of patients enrolled in a widely available telehealth obesity management program (WeightWatchers (WW) Clinic) that combines the provision of incretin‐based anti‐obesity medications (e.g., GLP‐1 and GLP‐1/GIP receptor agonists) with a behavioral program tailored for those on long‐acting incretin therapy. The effects of this program on weight loss, blood pressure, dietary quality, and physical activity have been previously reported [14]. The sample consisted of 180 consecutively enrolled participants (February—April 2024) who were prescribed either semaglutide or tirzepatide. Once the telehealth medical prescriber evaluated the patients for medical qualification and accepted them into the medical weight management program and prescribed either semaglutide or tirzepatide, they were invited to participate in the study, which included a virtual behavioral weight management program tailored for those long‐acting incretin therapy and online psychosocial assessments at baseline, and 12‐ and 24‐weeks post‐intervention. The study team did not determine eligibility for medication treatment (i.e., only those already approved for medication were eligible for enrollment). However, the study team confirmed inclusion and exclusion criteria prior to enrollment into the study:

Inclusion criteria:

1. 18 years or older
2. BMI of ≥ 30 kg/m^2^ or BMI of ≥ 27 kg/m^2^ with one or more weight related medically qualifying condition (hypertension, dyslipidemia, sleep apnea, cardiovascular disease)

Exclusion criteria:

1. Diabetes
2. Personal or family history of medullary thyroid carcinoma or Multiple Endocrine Neoplasia type 2
3. History of pancreatitis within 180 days
4. Previous surgical obesity treatment
5. Use of other anti‐obesity medications in the last 90 days
6. Use of a GLP‐1 within the last 180 days
7. Lost weight > 11 lbs. in the last 90 days
8. Pregnant, breastfeeding, intends to become pregnant, has child‐bearing potential and not using a reliable birth control method.

### 2.2 Intervention

Patients in this single‐arm study were enrolled in the WW Clinic. Within this clinic, potential participants first completed an online questionnaire to evaluate if they medically qualify for medical treatment of obesity. These guidelines align with those of the FDA for anti‐obesity medications (i.e., BMI ≥ 30 kg/m^2^ or 27 30 kg/m^2^ with an obesity related comorbidity). Once providing weight verification and considered a potential candidate, patients met virtually with a board‐certified clinician with additional training in obesity medicine. The clinician assessed if the patient was an appropriate candidate for AOMs and, if so, which ones would be most beneficial. In the current study, only participants prescribed semaglutide or tirzepatide were offered enrollment.

The WW Clinic includes access to a variety of obesity providers including registered dietitians, fitness specialists, care coordinators and a behavioral program that is tailored for those taking long‐acting incretin‐based AOMs. The behavioral program includes daily personalized nutritional goals related to protein, fruits and vegetables, and water to optimize intake of key vitamins, minerals and fiber. Activity goals are included to help attenuate lean muscle mass. Enrolled participants were provided with weekly, coach‐led virtual workshops focused on behavior change techniques and were given an app that included food, activity, water, weight trackers, a GLP‐1 Go‐To Foods list, meal planning tools, recipes and a members‐only social community with 24/7 access to chat with a coach. Additional information about the telemedicine program has been previously described [13, 14].

### 2.3 Study Procedures

Once determined to be medically qualified and enrolled by the clinic, all consecutive potential patients were offered the opportunity to take part in this study. The study team then pre‐screened interested participants via a virtual platform. Participants were eConsented and it was emphasized that the study was completely voluntary and that it would not affect their care within WW Clinic. Participants then completed the study questionnaires. Participants were instructed to sign up for one of the weekly virtual workshop offerings tailored for those on long‐acting incretin therapy within the WW app.

The WW GLP1 companion program includes personalized daily nutritional targets to optimize protein, fruit and vegetable consumption and water. This is designed to prioritize key vitamins, minerals, fiber and hydration. To maximize lean muscle mass, daily activity goals are provided. Participants in this trial were provided with weekly, 30–60 min, live, coach‐led virtual workshops focused on behavior change techniques and were given the WW GLP1 companion program app. The app supports self‐monitoring and includes food, activity, water, and weight trackers aided by a barcode scanner and available device connections. In addition, participants were able to access a GLP‐1 Go‐to Foods list and other resources in the What to Eat tab, guided meditations, always‐on support from peers via Connect (a members‐only social community), and WW‐trained behavior change experts via 24/7 chat with a Coach. The workshop schedule remained consistent week to week, but the curriculum and content changed weekly. Additional information about the telemedicine program has been previously described [13, 14].

The prescribing clinician determined all dosing adjustments and were guided by both BMI and the rate of weight loss with the following protocolized guidelines: (a) target weight loss rate was set at 2%–4% per month, with weight loss intended to not exceed 5% monthly; (b) as BMI approached 24–25, clinicians began assessing for dose maintenance or de‐escalation; (c) if weight loss fell below 0.5% per week (or 2% monthly), when weekly weight loss was 0.5%–0.75% (2%–3% monthly), the current dose was maintained until BMI reached 23; (d) if loss was 0.75%–1% per week (3%–4% monthly), dose de‐escalation by one step was recommended; (e) weight loss exceeding 1% per week (4%–6% monthly) warranted a two‐step dose reduction; (f) at BMI 22–24, the goal was to maintain weight on the lowest effective dose; and (g) if BMI dropped further, abrupt discontinuation was not required; instead, gradual dose reduction or extension of injection intervals was advised.

All visits were remote, and the study was approved by the Cleveland Clinic's Institutional Review Board (#23‐814) and registered on clinicaltrials.gov (NCT06034457).

### 2.4 Measures

The following measures were assessed at baseline, 12 and 24 weeks.

#### 2.4.1 Demographics

Age, sex assigned at birth, gender identity, marital status, education, ethnicity, race, household composition, household income, and social status (MacArthur Scale of Subjective Social Status) [15] were assessed via questions delivered electronically via REDCap.

#### 2.4.2 Clinical

Weight was measured utilizing a Conair Bluetooth enabled scale and systolic and diastolic blood pressure was measured using a Bluetooth enabled device (Withings Inc. BPM Connect Pro) mailed to their home. These results are reported elsewhere [14].

Additionally, participant engagement with the virtual behavioral program was measured via passive collection in the WW app. Participants also completed a monthly medication adherence questionnaire reporting any missed weekly doses and the reason for missing the dose. Participants received requests to upload their weight and blood pressure readings and to complete their surveys via email 7 days before the target data completion date. If they had not completed it by the target date, they received email reminders. The visit window aimed for −7 days to + 7 days of target date at 0, 12, and 24 weeks.

*Psychosocial Measures*: The following measures were utilized to assess diet and physical activity:

*Patient Health Questionnaire‐8* (PHQ‐8) [16]. The PHQ‐8 is a depression screening tool that was developed from the Patient Health Questionnaire‐9 [17] and omits the item assessing suicidality. It is often used when data are being gathered by self‐report rather than clinical interviews because follow‐up probing is not possible. The PHQ measures were designed to assess depressive symptoms that aligned with the DSM‐IV‐TR criteria [18].

*Perceived Stress Scale* (PSS‐10) [19]. PSS‐10 is a 10‐item self‐report measure that queries the extent to which events in life are perceived to be stressful. The items focus on more general life stressors rather than specific events and were designed for use in community samples.

*Self‐Compassion Scale* (SCS) [20]. The SCS is a 26‐item self‐report measure that assesses an individual's compassion toward themselves when experiencing perceived failure, suffering or insecurity.

*World Health Organization Well‐Being Index 5* (WHO‐5) [21]. WHO‐5 is a 5‐item self‐report instrument measuring mental well‐being. Each item focuses on the last 2 weeks with higher scores indicating better mental well‐being.

*The Two Factor Weight Bias Internalization Scale* (WBIS‐2F) [22]. The WBIS‐2F measures the weight‐related self‐stigma by assessing the degree to which an individual endorses whether negative stereotypes and self‐statements about persons with overweight and obesity apply to him or herself.

*Impact of Weight on Quality of Life‐Lite* (IWQOL‐Lite) [23]. The IWQOL‐Lite is a 31‐item self‐report measure of the quality of life specific to populations with obesity. It assesses 5 domains: physical function, self‐esteem, sexual life, public distress and work. For the current study, transformed scores were utilized and higher scores are associated with a greater negative impact of weight on quality of life.

### 2.5 Safety Monitoring

Adverse events and side effects related to the study drug were monitored by the WW Clinic provider during monthly clinical care visits and through ad hoc asynchronous communication. Participants were instructed to inform their PCP provider of any other adverse events or side effects unrelated to AOMs.

### 2.6 Data Analytic Plan

A sample size of 180 patients was selected to provide a representative assessment of psychosocial outcomes within this single arm study. All statistical analyses were performed using R statistical software (v.4.2.2) [24]. Intent‐to‐treat analysis was performed using all available data from enrolled participants. Imputation for study withdrawals (*n* = 14; 92.2% retention rate) and other missing data was performed using last observation carried forward (LOCF). Data were captured from patient‐completed surveys. Endpoints were summarized using descriptive statistics, and changes over time were assessed using Wilcoxon signed‐rank tests. Changes in study measures were evaluated between three intervals: baseline to 12 weeks, 12 weeks to 24 weeks, and baseline to 24 weeks. Statistics for continuous measures included patient counts (N), mean, SD, median, and interquartile range (IQR). Binary and categorical measures were summarized using frequencies and percentages. All reported *p* values have been adjusted for multiple testing using the Benjamini Hochberg false discovery rate (FDR) approach. An FDR‐adjusted *p* < 0.05 was used as the threshold for statistical significance. No adjustments for covariates were made.

## 3 Results

**FIGURE 1:**
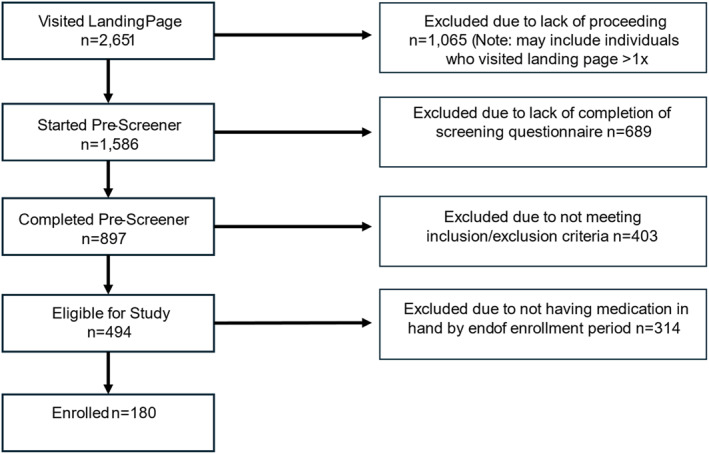
Enrollment flowchart.

Participants (*N* = 180) had an average baseline weight of 102.6 kg (SD = 20.9) and BMI of 37.13 kg/m^2^ (SD = 6.66). They averaged 44.1 years of age (SD = 10.4) and predominantly identified their sex and gender as female (91.1% and 90.0% respectively). 81.1% of the sample self‐identified as White, 11.1% as Black, and 7.8% endorsed a Hispanic ethnicity. The study population was relatively well‐educated and had a higher SES. Overall retention was 92% over the 24‐week trial. Semaglutide was prescribed to 60 participants and 120 received tirzepatide. Two participants who enrolled were removed due to an ineligible medication (unknown compounded medication) and one started an AOM prior to starting their prescribed long‐acting AOM, 5 participants were consented but did not complete any additional questionnaires at any study time‐points. Finally, 8 participants dropped out during the study and were lost to follow‐up. Additional demographic and socio‐economic information about the sample is provided in Table 1, and a flowchart of enrollment is depicted in Figure 1.

**TABLE 1:** Demographics of participants.

Demographic/clinical variable	Mean	SD
Age (years)	44.11	10.41
Weight (kgs.)	102.84	20.90
BMI (kg/m^2^)	37.13	6.66
BMI class	**Count**	**Percentage**
Overweight (BMI > 27 & < 30 kg/m^2^)	16	8.89
Class 1	62	34.44
Class 2	55	30.56
Class 3	47	26.11
Sex at birth	**Count**	**Percentage**
Male	16	8.89
Female	164	91.11
Marital status	**Count**	**Percentage**
Married	116	64.44
Living with a partner to whom I'm not married	23	12.78
Single	26	14.44
Separated	1	0.56
Divorced	14	7.78
Widowed	0	0.00
Education	**Count**	**Percentage**
Some high school	0	0.00
High school degree/G.E.D./equivalent	4	2.22
Trade school or specialty training	3	1.67
Some college	29	16.11
Associate's degree	11	6.11
Bachelor's degree	54	30.00
Some graduate school	16	8.89
Master's degree	42	23.33
Professional degree or doctorate	21	11.67
Ethnicity‐Hispanic	**Count**	**Percentage**
Yes	14	7.78
No	166	92.22
Race	**Count**	**Percentage**
American Indian or Alaska Native	2	1.11
Asian	2	1.11
Black or African American	20	11.11
Native Hawaiian or Pacific Islander	1	0.56
White	146	81.11
Other not listed	4	2.22
Prefer not to say	0	0.00
Unknown	1	0.56
Mixed race	4	2.22
Household income	**Count**	**Percentage**
Less than $50,000	6	3.33
$50,000 to $59,999	8	4.44
$60,000 to $69,999	8	4.44
$70,000 to $79,999	11	6.11
$80,000 to $89,999	8	4.44
$90,000 to $99,999	8	4.44
$100,000 to $149,999	51	28.33
$150,000 or more	80	44.44
SES ladder number (American Society)	**Count**	**Percentage**
Level 1	0	0.00
Level 2	0	0.00
Level 3	6	3.61
Level 4	15	9.04
Level 5	26	15.66
Level 6	46	27.71
Level 7	40	24.10
Level 8	23	13.86
Level 9	9	5.42
Level 10	1	0.60

Participants' self‐reported depressive symptoms on the PHQ‐8 significantly declined from baseline to 12 weeks and 24 weeks (all *p*'s < 0.0001). The decline from 12 to 24 weeks was not significant (*p* = 0.127). Perceived stress also waned from baseline to 12 weeks and to 24 week (all *p*'s < 0.0001). Perceived stress reduction from 12 to 24 weeks was not significant (*p* = 0.10).

Self‐compassion improved significantly from baseline to 12 weeks and to 24 weeks (*p <* 0.001). Self‐compassion also improved from 12 to 24 weeks (*p* < 0.001). Perceived well‐being as measured by the WHO‐5 raw scores significantly increased from baseline to 12 weeks and 24 weeks (all *p*'s < 0.0001). The improvement from 12 to 24 weeks was also significant (*p* < 0.002). The WHO‐5 percentage scores followed a similar pattern. Similarly, weight bias internalization significantly reduced from baseline to 12 weeks and 24 weeks (all *p*'s < 0.0001). The improvement of the 12 to 24 weeks was also significant (*p* < 0.0001).

Finally, IWQOL‐Lite total scores significantly improved from baseline to 12 weeks and 24 weeks (all *p*'s < 0.0001) and the 12‐ and 24‐week time‐points were also significant (*p* < 0.0001). Each of the IWQOL‐Lite subscales followed the same pattern. The medians, inter‐quartile range (IQR), absolute change and significance values for all measures can be found in Tables 2, 3, 4.

**TABLE 2:** Psychosocial outcome variables baseline to 12 weeks.

Measure	Baseline median (IQR)	12‐weeks median (IQR)	Absolute change median (IQR)	FDR *p* value
PHQ8 total score	5 (3, 9)	3 (2, 6)	−2 (−4, 0)	2.74 × 10^−11^
PSS‐10 total score	18 (13, 22)	15 (11, 20)	−2 (−6, 1)	1.73 × 10^−05^
Self‐compassion scale	2.9 (2.5, 3.3)	3.0 (2.6, 3.5)	0.1 (−0.2, 0.4)	8.15 × 10^−04^
WHO‐5 raw score	13 (10, 15)	15 (13, 18)	2 (0, 4)	4.41 × 10^−11^
WHO‐5 percentage score	52 (40, 60)	60 (52, 72)	8 (0, 16)	4.41 × 10^−11^
Weight bias internalization scale	3.6 (2.9, 4.5)	3.1 (2.5, 4.0)	−0.5 (−0.9, 0.0)	5.05 × 10^−12^
IWQOL‐lite transformed physical function	41 (24.5, 55)	25 (11, 39)	−12 (−23, −2)	2.25 × 10^−17^
IWQOL‐lite transformed self esteem	75 (57, 93)	54 (39, 75)	−14 (−28, 2.3)	4.55 × 10^−19^
IWQOL‐lite transformed sexual life	50 (25, 69)	38 (12, 56)	−6 (−25, 0)	3.37 × 10^−08^
IWQOL‐lite transformed public distress	25 (5, 40)	15 (0, 35)	−5 (−20, 0)	1.39 × 10^−07^
IWQOL‐lite transformed work	19 (0, 38)	12 (0, 26.5)	−3 (−19, 0)	1.31 × 10^−06^
IWQOL‐lite transformed total score	44 (30, 54)	31 (19.8, 44)	−11 (−19.3, −4)	4.15 × 10^−20^

**TABLE 3:** Psychosocial outcome variables baseline to 24 weeks.

Measure	Baseline median (IQR)	24‐weeks median (IQR)	Absolute change median (IQR)	FDR *p* value
PHQ8 total score	5 (3, 9)	3 (1, 6)	−2 (−5, 0)	1.96 × 10^−11^
PSS‐10 total score	18 (13, 22)	15 (11, 19.3)	−3 (−7, 1.25)	1.81 × 10^−07^
Self‐compassion scale	2.9 (2.5, 3.3)	3.1 (2.8, 3.6)	0.3 (−0.1, 0.7)	3.42 × 10^−09^
WHO‐5 raw score	13 (10, 15)	17 (13, 18)	3 (1, 5)	6.69 × 10^−16^
WHO‐5 percentage score	52 (40, 60)	68 (52, 72)	12 (4, 20)	6.69 × 10^−16^
Weight bias internalization scale	3.6 (2.9, 4.5)	2.9 (2.2, 3.7)	−0.6 (−1.3, 0.1)	2.02 × 10^−17^
IWQOL‐lite transformed physical function	41 (24.5, 55)	16 (7, 32)	−20 (−34, −7)	1.24 × 10^−23^
IWQOL‐lite transformed self esteem	75 (57, 93)	39 (25, 61.8)	−28 (−43, −13.3)	1.62 × 10^−25^
IWQOL‐lite transformed sexual life	50 (25, 69)	25 (0, 50)	−19 (−31, 0)	2.41 × 10^−17^
IWQOL‐lite transformed public distress	25 (5, 40)	10 (0, 26.3)	−10 (−20, 0)	8.30 × 10^−14^
IWQOL‐lite transformed work	19 (0, 38)	6 (0, 25)	−6 (−25, 0)	1.18 × 10^−12^
IWQOL‐lite transformed total score	44 (30, 54)	24 (12.8, 36.3)	−18 (−29, −10)	2.93 × 10^−27^

**TABLE 4:** Psychosocial outcome variables 12 weeks to 24 weeks.

Measure	12‐weeks median (IQR)	24‐weeks median (IQR)	Absolute change median (IQR)	FDR *p* value
PHQ8 total score	3 (2, 6)	3 (1, 6)	0 (−2, 1)	0.127
PSS‐10 total score	15 (11, 20)	15 (11, 19.3)	0 (−3, 2)	0.099
Self‐compassion scale	3.0 (2.6, 3.5)	3.1 (2.8, 3.6)	0.1 (−0.1, 0.4)	3.11 × 10^−04^
WHO‐5 raw score	15 (13, 18)	17 (13, 18)	0 (−1, 3)	1.19 × 10^−03^
WHO‐5 percentage score	60 (52, 72)	68 (52, 72)	0 (−4, 12)	1.19 × 10^−03^
Weight bias internalization scale	3.1 (2.5, 4.0)	2.9 (2.2, 3.7)	−0.2 (−0.7, 0.1)	3.01 × 10^−06^
IWQOL‐lite transformed physical function	25 (11, 39)	16 (7, 32)	−4 (−14, 0)	2.25 × 10^−11^
IWQOL‐lite transformed self esteem	54 (39, 75)	39 (25, 61.8)	−10 (−22, 0)	5.56 × 10^−14^
IWQOL‐lite transformed sexual life	38 (12, 56)	25 (0, 50)	−6 (−19, 0)	1.93 × 10^−08^
IWQOL‐lite transformed public distress	15 (0, 35)	10 (0, 26.3)	0 (−10, 0)	1.29 × 10^−04^
IWQOL‐lite transformed work	12 (0, 26.5)	6 (0, 25)	0 (−12.3, 0)	1.29 × 10^−04^
IWQOL‐lite transformed total score	31 (19.8, 44)	24 (12.8, 36.3)	−6 (−12, 0)	8.12 × 10^−16^

## 4 Discussion

This pragmatic single‐arm, prospective study demonstrates an association between semaglutide and tirzepatide administered via telemedicine combined with a virtually delivered behavioral intervention program tailored for those on long‐acting incretin therapy and a number of psychosocial domains. Consistent with many current practices, participants received both pharmacological and behavioral interventions entirely via a virtual telehealth platform.

Over the course of the trial, depression, stress and perceived well‐being significantly improved. This depression finding is consistent with the carefully controlled, in‐person randomized clinical trials of semaglutide and tirzepatide demonstrating significant declines in depressive symptoms [25]. Similar to those studies, the participants in the current trial only endorsed mild depressive symptoms at baseline. However, the declines are promising, particularly given concerns about psychiatric side effects such as increased suicidal ideation shown in some studies [6, 26]. Relatedly, the current sample reported baseline perceived stress that was a standard deviation above population norms. Although the median after 24 weeks remained higher than the norm, it fell within the norm's interquartile range [27]. Although previously investigated in behavioral weight loss interventions [28], past efficacy investigations of semaglutide and tirzepatide have not examined perceived well‐being, a construct often associated with depression [29]. Unlike the behavioral intervention literature, the cohort studied demonstrated significant improvements from baseline to 12 weeks and 12 to 24 weeks, and at that latter time‐point, their perceived well‐being was consistent with population norms [30]. Given the greater weight loss experienced by those on AOMs, the improvements in perceived well‐being may relate to the change in obesity more than skills developed in the paired behavioral intervention. However, identifying the mechanism would be an interesting avenue for future research. Similarly, perceived stress has not been examined in the context of the initiation of these AOMs. The results indicate significant declines at all time‐points although participants remained in the moderate range of perceived stress.

Baseline scores on weight bias internalization were similar to those previously reported in a treatment seeking (WW behavioral intervention) population with obesity. Weight bias internalization significantly improved from baseline at both 12 and 24 weeks and the improvements were shown from the intermediate to final assessment. It has been suggested that AOMs such as semaglutide and tirzepatide may reduce weight bias as they frame obesity as a disease instead of due to behaviors under the patients' control. However, it has also been argued that these AOMs—like metabolic and bariatric surgery—may be seen as “an easy way out” by patients and the lay public [31, 32]. These results suggest that the use of the medications paired with a relevant behavioral intervention helped reduce internalized bias. In contrast, self‐compassion did not demonstrate robust improvements. Only the baseline to 24‐week time‐points were associated with a small increase in self‐compassion. This is somewhat different from past studies examining self‐compassion in a behavioral weight loss intervention through WW. For example, baseline self‐compassion in this cohort was higher than previously reported in participants seeking a behavioral weight loss intervention through WW [33]. In a WW 6‐month trial examining the impact of the Points program, self‐compassion baseline scores were similar; however, self‐compassion improved 8.3% in the WW group (vs. 1.2% in the control) as compared to a 2% increase [34]. It is not clear if this may be the result of internalized assumptions about medications as noted above or if greater involvement in the behavioral intervention occurs when there is not paired medication.

This pragmatic, telehealth delivered study also found improvements in weight‐related quality of life, as has been shown in carefully controlled, in‐person randomized clinical trials [3, 4]. Beyond weight loss, patients receiving telehealth medication and medication‐specific virtual lifestyle intervention reported improvements in their overall quality of life as well as physical function, self‐esteem, sexual life, public distress and work life.

The current study is the first to demonstrate the benefit of a combined telemedicine AOM prescription with a comprehensive lifestyle program tailored for those on long‐acting incretin therapy on psychosocial outcomes. Previous trials have included monthly counseling on caloric reduction and increasing exercise. However, the current intervention provided much more comprehensive support including frequent treatment and interaction with the interventionist, group support, skill development focusing on behavioral principles, psychoeducation and facilitation by a variety of obesity experts. It is hypothesized that many of the psychosocial benefits reported were due to both weight loss as well as the virtual, tailored behavioral program (e.g., positive self‐talk, problem solving, stimulus control, etc.). Given that the majority of patients discontinue medications over time [35], the development of behavioral skills may improve psychosocial outcomes even after drug discontinuation although longer‐term randomized trials are needed to rigorously test that hypothesis.

The current study has a number of strengths. As previously noted, this pragmatic study is more aligned with the real‐world usage of incretin‐based AOMs and was prospectively designed. Unlike previous trials that have largely focused on weight loss and metabolic and cardiovascular outcomes, a wide variety of psychosocial outcomes were included. Finally, all the latest reported data were included in the intent‐to‐treat analyses to increase generalizability in examining real world experience with intervention.

The current study is limited by its single‐arm design that did not include a control group. Findings would be strengthened by the inclusion of a placebo or first generation AOMs as comparison groups. The design is also unable to separate the effect of the AOM from the impact of the behavioral intervention. Improvements in these psychosocial outcomes may be due to weight loss, skills developed during the behavioral intervention and/or biological effects of the medications themselves. Future randomized controlled trials that include arms that receive only AOMs and only receive the WW lifestyle intervention and the combination would allow for a more precise understanding of the benefit of each treatment component. Further, future work should explore the mediating role of both baseline BMI and percent total weight loss on the changes in psychosocial variables over time. This study was conducted during a period of significant drug shortages and a considerable subset of participants missed doses during the 24‐week trial due to difficulties filling their prescriptions. Although this reflects the real‐world experience of patients and intent‐to‐treat analyses were conducted to include those lost to follow‐up and those with missing weeks of medication, the results may have been attenuated due to these shortages. Additionally, although the number of participants that were lost to follow‐up was small (< 8%), this may have had an impact on the results. Because we were unable to determine whether participants maintained changes over time, we elected to utilize an LOCF approach, which assumes no change after being lost to follow up. However, it is possible that this method underestimated the benefits for individuals that continued to lose weight/implement behavioral skills learned during the intervention or overestimated the benefits for individuals with weight recurrence or reduced utilizing behavioral change strategies. The current sample was predominantly mid‐life, female, well‐educated and from higher socio‐economic groups. However, unlike the larger RCTs, this study had Black participants relatively equal to the US population. Future studies would benefit from a more diverse group of participants who reflect the population that is disproportionately impacted by obesity.

In conclusion, this novel prospective trial evaluated a real‐world, virtually delivered, comprehensive lifestyle‐based intervention tailored for those on long‐acting incretin therapy and focused on dietary intake, physical activity, support and behavioral principles in conjunction with telemedicine delivery of semaglutide/tirzepatide and demonstrated efficacy in improving depression, perceived stress, well‐being, weight bias internalization and weight‐related quality of life. Combined telemedicine with virtually delivered lifestyle interventions for obesity is a viable option for improving access to clinically significant outcomes, although longer term outcomes should be assessed.

## Funding

This study was supported by WW International Inc.

## Conflicts of Interest

A.M.L., G.D.F. and M.I.C. were employees of WW International Inc. during the majority of the work. M.I.C. is a shareholder of WW International Inc. A.M.L. is an employee and shareholder of Eli Lilly and Company. This work was completed prior to A.M.L.'s employment at Lilly. A.M.L. is acting entirely on her own, and this work is not affiliated with Lilly in any manner. L.J.H. received a research grant from WW International Inc.

## Data Availability

The data that support the findings of this study are available from the corresponding author upon reasonable request.

## References

[1] A. M. Jastreboff , L. J. Aronne , N. N. Ahmad , et al., “SURMOUNT‐1 Investigators. Tirzepatide Once Weekly for the Treatment of Obesity,” New England Journal of Medicine 387, no. 3 (July 2022): 205–216: Epub 2022 Jun 4. PMID: 35658024, 10.1056/NEJMoa2206038. 35658024

[2] D. M. Rubino , F. L. Greenway , U. Khalid , et al., “STEP 8 Investigators. Effect of Weekly Subcutaneous Semaglutide vs Daily Liraglutide on Body Weight in Adults With Overweight or Obesity Without Diabetes: The STEP 8 Randomized Clinical Trial,” JAMA 327, no. 2 (January 2022): 138–150, 10.1001/jama.2021.23619. 35015037 PMC8753508

[3] D. Rubino , J. B. Bjorner , N. Rathor , et al., “Effect of Semaglutide 2.4 Mg on Physical Functioning and Weight‐ and Health‐Related Quality of Life in Adults With Overweight or Obesity: Patient‐Reported Outcomes From the STEP 1‐4 Trials,” Diabetes, Obesity and Metabolism 26, no. 7 (July 2024): 2945–2955: Epub 2024 Mays, 10.1111/dom.15620. 38698650

[4] K. A. Gudzune , A. Stefanski , D. Cao , et al., “Association Between Weight Reduction Achieved With Tirzepatide and Quality of Life in Adults With Obesity: Results From the SURMOUNT‐1 Study,” Diabetes, Obesity and Metabolism 27, no. 2 (February 2025): 539–550, 10.1111/dom.16046. PMC11701187 39497468

[5] X. Chen , P. Zhao , W. Wang , L. Guo , and Q. Pan , “The Antidepressant Effects of GLP‐1 Receptor Agonists: A Systematic Review and Meta‐Analysis,” American Journal of Geriatric Psychiatry 32, no. 1 (January 2024): 117–127, 10.1016/j.jagp.2023.08.010. 37684186

[6] G. Schoretsanitis , S. Weiler , C. Barbui , E. Raschi , and C. Gastaldon , “Disproportionality Analysis From World Health Organization Data on Semaglutide, Liraglutide, and Suicidality,” JAMA Network Open 7, no. 8 (August 2024): e2423385, 10.1001/jamanetworkopen.2024.23385. 39163046 PMC11337067

[7] E. Kornelius , J. Y. Huang , S. C. Lo , C. N. Huang , and Y. S. Yang , “The Risk of Depression, Anxiety, and Suicidal Behavior in Patients With Obesity on Glucagon Like Peptide‐1 Receptor Agonist Therapy,” Scientific Reports 14, no. 1 (October 2024): 24433, 10.1038/s41598-024-75965-2. 39424950 PMC11489776

[8] W. Wang , N. D. Volkow , N. A. Berger , P. B. Davis , D. C. Kaelber , and R. Xu , “Association of Semaglutide With Risk of Suicidal Ideation in a Real‐World Cohort,” Nature Medicine 30, no. 1 (January 2024): 168–176, 10.1038/s41591-023-02672-2. PMC11034947 38182782

[9] R. S. McIntyre , R. B. Mansur , J. D. Rosenblat , et al., “Glucagon‐Like Peptide‐1 Receptor Agonists (GLP‐1 RAs) and Suicidality: A Replication Study Using Reports to the World Health Organization Pharmacovigilance Database (VigiBase®),” Journal of Affective Disorders 369 (January 2025): 922–927, 10.1016/j.jad.2024.10.062. 39433133

[10] A. Montero , G. Sparks , A. Kirzinger , I. Valdes , and L. Hamel , KFF Health Tracking Poll July 2023: The Public’s Views of New Prescription Weight Loss Drugs and Prescription Drug Costs (2023).

[11] M. L. Griebeler , W. S. Butsch , P. Rodriguez , et al., “The Use of Virtual Visits for Obesity Pharmacotherapy in Patients With Overweight or Obesity Compared With In‐Person Encounters,” Obesity 30, no. 11 (2022): 2194–2203, 10.1002/oby.23548. 36156456 PMC9826334

[12] S. Kahan , M. Look , and A. Fitch , “The Benefit of Telemedicine in Obesity Care,” Obesity 30, no. 3 (2022): 577–586, 10.1002/oby.23382. 35195367

[13] J. D. Ard , Y. R. Hong , G. D. Foster , A. Medcalf , S. Nadolsky , and M. I. Cardel , “Twelve‐Month Analysis of Real‐World Evidence From a Telehealth Obesity‐Treatment Provider Using Antiobesity Medications,” Obesity 32, no. 12 (December 2024): 2246–2254, 10.1002/oby.24169. 39478294 PMC11589532

[14] L. J. Heinberg , A. M. Lee , G. D. Foster , et al., “Effectiveness of Telemedicine Prescribing and a Long‐Acting Obesity Medication Behavioral Program: A 24‐Week Single‐Arm Study,” Obesity 18 (November 2025): oby.70056: Epub ahead of print. PMID: 41253738, 10.1002/oby.70056. 41253738

[15] N. E. Adler , E. S. Epel , G. Castellazzo , and J. R. Ickovics , “Relationship of Subjective and Objective Social Status With Psychological and Physiological Functioning: Preliminary Data in Healthy White Women,” Health Psychology 19, no. 6 (November 2000): 586–592, 10.1037/0278-6133.19.6.586. 11129362

[16] K. Kroenke , T. W. Strine , R. L. Spitzer , J. B. Williams , J. T. Berry , and A. H. Mokdad , “The PHQ‐8 as a Measure of Current Depression in the General Population,” Journal of Affective Disorders 114, no. 1–3 (April 2009): 163–173, 10.1016/j.jad.2008.06.026. 18752852

[17] K. Kroenke , R. L. Spitzer , and J. B. W. Williams , “The PHQ‐9: Validity of a Brief Depression Severity Measure,” Journal of General Internal Medicine 16, no. 9 (2001): 606–613, 10.1046/j.1525-1497.2001.016009606.x. 11556941 PMC1495268

[18] American Psychiatric Association , Diagnostic and Statistical Manual of Mental Disorders, 4th ed. (1994).

[19] S. Cohen and G. Williamson , “Perceived Stress in a Probability Sample of the United States,” in The Social Psychology of Health: Claremont Symposium on Applied Social Psychology, eds. S. Spacapan and S. Oskamp (Sage, 1988).

[20] F. Raes , E. Pommier , K. D. Neff , and D. Van Gucht , “Construction and Factorial Validation of a Short Form of the Self‐Compassion Scale,” Clinical Psychology & Psychotherapy 18, no. 18 (2011): 250–255, 10.1002/cpp.702. 21584907

[21] World Health Organization , The World Health Organization‐Five Well‐Being Index (WHO‐5) (World Health Organization, 2024).

[22] A. Meadows and S. Higgs , “The Multifaceted Nature of Weight‐Related Self‐Stigma: Validation of the Two‐Factor Weight Bias Internalization Scale (WBIS‐2F),” Frontiers in Psychology 10 (April 2019): 808, 10.3389/fpsyg.2019.00808. 31040808 PMC6477068

[23] R. L. Kolotkin , R. D. Crosby , K. D. Kosloski , and G. R. Williams , “Development of a Brief Measure to Assess Quality of Life in Obesity,” Obesity Research 9, no. 2 (2001): 102–111, 10.1038/oby.2001.13. 11316344

[24] R Core Team , R: A Language and Environment for Statistical Computing (R Foundation for Statistical Computing, 2022).

[25] T. A. Wadden , G. K. Brown , C. Egebjerg , et al., “Psychiatric Safety of Semaglutide for Weight Management in People Without Known Major Psychopathology: Post Hoc Analysis of the STEP 1, 2, 3, and 5 Trials,” JAMA Internal Medicine 184, no. 11 (November 2024): 1290–1300, 10.1001/jamainternmed.2024.4346. 39226070 PMC11372653

[26] K. Valentino , K. M. Teopiz , W. Cheung , et al., “The Effect of Glucagon‐Like Peptide‐1 Receptor Agonists on Measures of Suicidality: A Systematic Review,” Journal of Psychiatric Research 183 (March 2025): 112–126, 10.1016/j.jpsychires.2025.02.008. 39956093

[27] F. Huang , H. Wang , Z. Wang , et al., “Psychometric Properties of the Perceived Stress Scale in a Community Sample of Chinese,” BMC Psychiatry 20, no. 1 (March 2020): 130: Erratum in: BMC Psychiatry. 2020 May 25;20(1):260. doi: 10.1186/s12888‐020‐02670‐5. PMID: 32197589; PMCID: PMC7082906, 32197589 10.1186/s12888-020-02520-4 PMC7082906

[28] R. A. Jones , E. R. Lawlor , J. M. Birch , et al., “The Impact of Adult Behavioural Weight Management Interventions on Mental Health: A Systematic Review and Meta‐Analysis,” Obesity Reviews 22, no. 4 (2021): e13150, 10.1111/obr.13150. 33103340 PMC7116866

[29] C. W. Topp , S. D. Østergaard , S. Søndergaard , and P. Bech , “The WHO‐5 Well‐Being Index: A Systematic Review of the Literature,” Psychotherapy and Psychosomatics 84, no. 3 (2015): 167–176, 10.1159/000376585. 25831962

[30] A. Domenech , I. Kasujee , V. Koscielny , and C. E. M. Griffiths , “Systematic Review of the Use of the WHO‐5 Well‐Being Index Across Different Disease Areas,” Advances in Therapy (June 2025). 10.1007/s12325-025-03266-9 PMC12313798 40506676

[31] R. L. Pearl , M. S. Himmelstein , R. M. Puhl , T. A. Wadden , A. C. Wojtanowski , and G. D. Foster , “Weight Bias Internalization in a Commercial Weight Management Sample: Prevalence and Correlates,” Obesity Science and Practice 5, no. 4 (July 2019): 342–353, 10.1002/osp4.354. 31452919 PMC6700514

[32] B. L. Heitmann , “The Impact of Novel Medications for Obesity on Weight Stigma and Societal Attitudes: A Narrative Review,” Current Obesity Reports 14, no. 1 (February 2025): 18, 10.1007/s13679-025-00611-5. 39907856 PMC11799028

[33] J. M. Brenton‐Peters , N. S. Consedine , A. Cavadino , R. Roy , K. H. Ginsberg , and A. Serlachius , “Finding Kindness: A Randomized Controlled Trial of an Online Self‐Compassion Intervention for Weight Management (SC4WM),” British Journal of Health Psychology 29, no. 1 (2024): 37–58, 10.1111/bjhp.12686. 37544883

[34] A. M. Palacios , M. I. Cardel , C. Q. Watts , et al. “Effectiveness of a Digital Weight Management Program on Weight‐Bias Internalization, Self‐Compassion, Quality of Life, Well‐Being, and Body Appreciation: Secondary Outcomes From a Randomized Controlled Trial.” Annals of Behavioral Medicine (forthcoming).

[35] P. P. Gleason , B. Y. Urick , L. Z. Marshall , N. Friedlander , Y. Qiu , and R. S. Leslie , “Real‐World Persistence and Adherence to Glucagon‐Like Peptide‐1 Receptor Agonists Among Obese Commercially Insured Adults Without Diabetes,” Journal of Managed Care & Specialty Pharmacy 30, no. 8 (August 2024): 860–867, 10.18553/jmcp.2024.23332. 38717042 PMC11293763

