# Correction to “Psychosocial Outcomes in Telemedicine and Long‐Acting Incretin‐Specific Behavioral Intervention”

**DOI:** 10.1002/osp4.70190

**Published:** 2026-08-29

L. J. Heinberg, A. M. Lee, G. D. Foster, et al., “Psychosocial Outcomes in Telemedicine and Long‐Acting Incretin‐Specific Behavioral Intervention,” *Obesity Science & Practice* 12, no. 3 (May 2026): e70149, PMID: 42256457; PMCID: PMC13240154, https://doi.org/10.1002/osp4.70149.

Our reporting of the *Impact of Weight on Quality of Life‐Lite* (IWQOL‐Lite) data may lead to confusion. We noted in the paper that “for the current study, transformed scores were utilized and higher scores are associated with a greater negative impact of weight on quality of life”. The raw scores were transformed but were reported such that higher scores are associated with a greater impact of obesity on quality of life. However, as the measure's authors recommend, a higher score means *less* impact of weight on quality of life. Although there aren’t logical inconsistencies if someone reads the paper carefully, we are concerned that it could lead to confusion. Thus, we are changing the tables and altering the sentence on the directionality in the “Methods” section.

Tables 2, 3, 4 are now updated and placed below (please note *p*‐values are unchanged and we confirmed all calculations). We also shortened the sentence in the methods to say, “for the current manuscript, transformed scores were utilized.”

**TABLE 2:** Psychosocial outcome variables baseline to 12‐weeks.

Measure	Baseline median (IQR)	12‐weeks median (IQR)	Absolute change median (IQR)	FDR *p* value
PHQ8 total score	5 (3, 9)	3 (2, 6)	−2 (−4, 0)	2.74 × 10^−11^
PSS‐10 total score	18 (13, 22)	15 (11, 20)	−2 (−6, 1)	1.73 × 10^−05^
Self‐Compassion Scale	2.9 (2.5, 3.3)	3.0 (2.6, 3.5)	0.1 (−0.2, 0.4)	8.15 × 10^−04^
WHO‐5 Raw Score	13 (10, 15)	15 (13, 18)	2 (0, 4)	4.41 × 10^−11^
WHO‐5 Percentage Score	52 (40, 60)	60 (52, 72)	8 (0, 16)	4.41 × 10^−11^
Weight Bias Internalization Scale	3.6 (2.9, 4.5)	3.1 (2.5, 4.0)	−0.5 (−0.9, 0.0)	5.05 × 10^−12^
IWQOL‐Lite transformed Physical Function	59 (45, 75.5)	75 (61, 89)	12 (2, 23)	2.25 × 10^−17^
IWQOL‐Lite transformed Self Esteem	25 (7, 43)	46 (25, 61)	14 (2.3, 28)	4.55 × 10^−19^
IWQOL‐Lite transformed Sexual Life	50 (31, 75)	62 (44, 88)	6 (0, 25)	3.37 × 10^−08^
IWQOL‐Lite transformed Public Distress	75 (60, 95)	85 (65, 100)	5 (0, 20)	1.39 × 10^−07^
IWQOL‐Lite transformed Work	81 (62, 100)	88 (73.5, 100)	3 (0, 19)	1.31 × 10^−06^
IWQOL‐Lite transformed Total Score	56 (46, 70)	69 (56, 80.3)	11 (4, 19.3)	4.15 × 10^−20^

**TABLE 3:** Psychosocial outcome variables baseline to 24‐weeks.

Measure	Baseline median (IQR)	24‐weeks median (IQR)	Absolute change median (IQR)	FDR *p* value
PHQ8 total score	5 (3, 9)	3 (1, 6)	−2 (−5, 0)	1.96 × 10^−11^
PSS‐10 total score	18 (13, 22)	15 (11, 19.3)	−3 (−7, 1.25)	1.81 × 10^−07^
Self‐Compassion Scale	2.9 (2.5, 3.3)	3.1 (2.8, 3.6)	0.3 (−0.1, 0.7)	3.42 × 10^−09^
WHO‐5 Raw Score	13 (10, 15)	17 (13, 18)	3 (1, 5)	6.69 × 10^−16^
WHO‐5 Percentage Score	52 (40, 60)	68 (52, 72)	12 (4, 20)	6.69 × 10^−16^
Weight Bias Internalization Scale	3.6 (2.9, 4.5)	2.9 (2.2, 3.7)	−0.6 (−1.3, 0.1)	2.02 × 10^−17^
IWQOL‐Lite transformed Physical Function	59 (45, 75.5)	84 (68, 93)	20 (7, 34)	1.24 × 10^−23^
IWQOL‐Lite transformed Self Esteem	25 (7, 43)	61 (38.3, 75)	28 (13.3, 43)	1.62 × 10^−25^
IWQOL‐Lite transformed Sexual Life	50 (31, 75)	75 (50, 100)	19 (0, 31)	2.41 × 10^−17^
IWQOL‐Lite transformed Public Distress	75 (60, 95)	90 (73.8, 100)	10 (0, 20)	8.30 × 10^−14^
IWQOL‐Lite transformed Work	81 (62, 100)	94 (75, 100)	6 (0, 25)	1.18 × 10^−12^
IWQOL‐Lite transformed Total Score	56 (46, 70)	76 (63.8, 87.3)	18 (10, 29)	2.93 × 10^−27^

**TABLE 4:** Psychosocial outcome variables 12‐ to 24‐weeks.

Measure	12‐weeks median (IQR)	24‐weeks median (IQR)	Absolute change median (IQR)	FDR *p* value
PHQ8 total score	3 (2, 6)	3 (1, 6)	0 (−2, 1)	0.127
PSS‐10 total score	15 (11, 20)	15 (11, 19.3)	0 (−3, 2)	0.099
Self‐Compassion Scale	3.0 (2.6, 3.5)	3.1 (2.8, 3.6)	0.1 (−0.1, 0.4)	3.11 × 10^−04^
WHO‐5 Raw Score	15 (13, 18)	17 (13, 18)	0 (−1, 3)	1.19 × 10^−03^
WHO‐5 Percentage Score	60 (52, 72)	68 (52, 72)	0 (−4, 12)	1.19 × 10^−03^
Weight Bias Internalization Scale	3.1 (2.5, 4.0)	2.9 (2.2, 3.7)	−0.2 (−0.7, 0.1)	3.01 × 10^−06^
IWQOL‐Lite transformed Physical Function	75 (61, 89)	84 (68, 93)	4 (0, 14)	2.25 × 10^−11^
IWQOL‐Lite transformed Self Esteem	46 (25, 61)	61 (38.3, 75)	10 (0, 22)	5.56 × 10^−14^
IWQOL‐Lite transformed Sexual Life	62 (44, 88)	75 (50, 100)	6 (0, 19)	1.93 × 10^−08^
IWQOL‐Lite transformed Public Distress	85 (65, 100)	90 (73.8, 100)	0 (0, 10)	1.29 × 10^−04^
IWQOL‐Lite transformed Work	88 (73.5, 100)	94 (75, 100)	0 (0, 12.3)	1.29 × 10^−04^
IWQOL‐Lite transformed Total Score	69 (56, 80.3)	76 (63.8, 87.3)	6 (0, 12)	8.12 × 10^−16^

We apologize for this error.

